# Hospital-acquired urinary tract infection: a case control study

**DOI:** 10.1186/1753-6561-5-S6-P201

**Published:** 2011-06-29

**Authors:** LN Markovic-Denic, B Mijovic, S Jankovic

**Affiliations:** 1 Institute of Epidemiology, School of Medicine, University of Belgrade, Belgrade, Serbia; 2 Institute of Public Health, Uzice, Serbia

## Introduction / objectives

Hospital-acquired urinary tract infections (HAUTIs) are responsible for about 40% of all healthcare-associated infections. The aim of the study was to assess risk factors and microbiological aspects of HAUTI on six wards of a general regional hospital in Serbia.

## Methods

A case-control study was nested within prospective cohort HAUTIs study conducted from January to December, 2007. Surveillance was performed on all patients admitted directly from the community to one of the study wards and whose hospital stay covered 72 h or more. The cases were patients with HAUTIs, identified using definition of the Centers for Disease Control and Prevention. Three controls were identified for each case, being chronologically the next three patients surveyed who did not develop HAUTI. The patients and controles were mached by sex and age (+5 years).

## Results

Assessment of 8,467 patients during study period revealed HAUTI in 125 of these. The overall incidence rate of HAUTI was 14.8 cases/1000 admissions. The mean age (range) of cases and controls were 64.9 (18-85) and 65.2 (17-86), retrospectively. Multivariate logistic regression analysis showed that increasing length of urinary catheterization (odds ratio [OR], 13.22; 95% CI, 3.36-51.91) and increasing length of hospitalisation (odds ratio [OR], 1.21; 95% CI, 1.04-1.42) were independently associated with increased risk of HAUTIs. The most frequently isolated Gram-negative bacteria were *Enterobacter*, *Klebsiella* sp, *Proteus mirabilis* and *Escherichia coli*. *Enterococcus sp* was the most frequent Gram-positive bacteria.

## Conclusion

The length of urinary catheterization and prolonged hospitalization were the most important risk factors of HAUTIs.

## Disclosure of interest

None declared.

